# Vomiting While under an Æsthetics

**Published:** 1897-10-16

**Authors:** 


### VOMITING WHILE UNDER ANÆSTHETICS.

The occurrence of a case of death while under an anæsthetic (gas and ether), due apparently to the inhalation of vomited matter, has led Mr. Bertram Hunt to discuss again the advisability of washing out the stomach in cases of intestinal obstruction requiring operation. Several dangers are apt to arise from leaving the stomach loaded in such cases. (*a*) The absorption of the poisonous material which accumulates in the stomach in most cases of obstruction, often causes severe toxic symptoms, and even death, some hours after the obstruction has been relieved by operation; (*b*) vomiting may occur, and the stercoral matter being inhaled into the lungs during anæsthesia, or even an hour or so after recovery from it, may cause death; (*c*) a minor degree of the same accident may set up severe or fatal septic pneumonia.

The late Mr. Greig Smith advised that in any case of intestinal obstruction with a distended stomach either the stomach should be washed out before giving the anæsthetic or a local anæsthetic only should be used There is, however, this disadvantage in such a proceeding, that the necessary manipulations in a conscious person often cause such retching that collapse may easily occur when a patient is already in a critical condition, and Mr. Bertram Hunt, balancing the advantages and disadvantages of the various courses open to the surgeon, concludes in favour of performing lavage as soon as the patient is fully under the anæsthetic, taking means to minimise, as far as possible, the danger from vomiting occurring before the stomach is emptied.

